# IGFBP1 is a prognostic biomarker in esophageal carcinoma: mechanistic insights from bioinformatics profiling to *in vitro* functional validation

**DOI:** 10.3389/fimmu.2026.1836824

**Published:** 2026-09-16

**Authors:** Huijuan Jiang, Junling Zhu, Zhang Lei, Tuxunayi Yimamu, Hailin Chen, Qiannan Li, Aishanjiang Muhetaer, Ainiwaerjiang Abudourousuli

**Affiliations:** 1 Department of Pathology, The First People’s Hospital of Kashi Prefecture, Kashi, China; 2 Department of Thoracic Surgery, The First People’s Hospital of Kashi Prefecture, Kashi, China; 3 School of Medicine, Kashi University, Kashi, China; 4 Institute of Etiology and Metabolic Diseases in Pamir Plateau Area, Kashi University, Kashi, China; 5 Kashi Prefecture Cancer Research Institute, The First People’s Hospital of Kashi Prefecture, Kashi, China

**Keywords:** biomarker, esophageal carcinoma, IGFBP1, prognosis, tumor microenvironment

## Abstract

Insulin-like growth factor binding protein 1 (IGFBP1) is documented as a pro- tumorigenic gene in various cancers; nevertheless, its specific function in esophageal carcinoma (ESCA) is unclear. This study used data obtained from The Cancer Genome Atlas (TCGA) to assess IGFBP1 expression, investigating its clinical outcome-related relevance, and explore its correlation in the context of ESCA tumor microenvironment. The Wilcoxon rank-sum test indicated a notable overexpression of IGFBP1 in ESCA compared to normal tissues, which was further validated via immunohistochemistry. Functional enrichment analysis identified tumor-related pathways, while ssGSEA/CIBERSORT methods linked IGFBP1 expression to altered immune infiltration. Furthermore, an investigation of single-cell data sourced from the GSE196756 data uncovered unique immune cell subsets linked to the expression of IGFBP1. The application of Cox regression alongside Kaplan-Meier survival analyses indicated that elevated levels of IGFBP1 were correlated with unfavorable outcomes in overall survival (OS), and disease-specific survival (DSS). Functional validation utilizing CCK-8 and transwell assays revealed that IGFBP1 silencing significantly suppressed the proliferative, invasive, and migratory capabilities of ESCA cell lines. Furthermore, *in vivo* analyses also presented similar results. Collectively, these results position IGFBP1 as a prognostic biomarker in ESCA, highlighting its functional involvement in tumor progression.

## Introduction

Esophageal carcinoma (ESCA) is classified as one of the most aggressive forms of cancer, with global statistics indicating more than 600,000 new diagnoses and approximately 540,000 fatalities in 2020 (1). This positions ESCA as the 8th most prevalent cancer and the 6th main contributor to cancer-associated deaths (2). From a histological perspective, ESCA is mainly categorized into two predominant subtypes: esophageal adenocarcinoma (EA) and esophageal squamous cell carcinoma (ESCC). Notably, the latter accounts for more than 90% of all diagnosed cases (3). The occurrence of ESCA shows considerable variation among different populations and geographic areas, highlighting the intricate interactions between genetic factors and environmental influences (4). ESCA incidence is driven by distinct risk factors, linked to tobacco use, alcohol consumption, obesity and chronic gastroesophageal reflux disease (5, 6). Current diagnostic methods rely on endoscopic biopsy with histopathological confirmation, supplemented by imaging modalities (CT/PET) for staging (7). Therapeutic strategies, including surgery, chemoradiation, and immune checkpoint inhibitors, have improved survival in localized disease (8). Nevertheless, over 60% of patients present with advanced-stage ESCA, for which 5-year survival is consistently below 20% (9). This dismal prognosis, compounded by the absence of reliable early biomarkers and frequent therapeutic resistance, underscores the urgent need for novel molecular targets to refine risk stratification and precision therapy. Insulin-like growth factor-binding protein 1 (IGFBP1), which is encoded by a gene situated on chromosome 7p12.3, serves as a versatile modulator of the IGF signaling pathway (10). As a high-affinity binder of IGF-I/II, IGFBP1 governs ligand-receptor interactions by modulating IGF bioavailability, tissue partitioning, and clearance, thereby exerting dual roles in suppressing or promoting tumorigenesis depending on cellular context (11–13). Beyond its canonical IGF-dependent functions, IGFBP1 drives cancer progression through IGF-independent mechanisms, including direct binding to integrins (e.g., α5β1) to activate FAK/SRC signaling, enhance epithelial-mesenchymal transition, and inhibit anoikis (14). These effects have established IGFBP1 as a context-dependent oncogene in breast cancer (pro-metastatic) (15), colorectal cancer (chemoresistance) (16), lung adenocarcinoma (17, 18) and hepatocellular carcinoma (hypoxia-driven angiogenesis) (19). Notably, recent studies implicate IGFBP1 in the RNA N6-methyladenosine (m6A) modification network, where it may coordinate with writers (e.g., METTL3) or readers (e.g., YTHDF2) to regulate mRNA stability and translation in tumors (20). Previous studies demonstrated that the IGFBP1^+^WNT3A^-^ biomarker, based on WNT signaling, effectively predicts the response and prognosis of ESCA patients to PD-L1 inhibitor treatment (21). Despite its well-characterized roles in other malignancies, the prognostic role of IGFBP1 remains understudied in ESCA. Preliminary reports suggest elevated serum IGFBP1 levels in ESCA patients (22), but its tissue-specific expression patterns, prognostic significance, and functional mechanisms-particularly its interplay with m6A modifiers, immune microenvironments, and direct tumor-promoting phenotypes like proliferation and invasion—are unexplored.

To address this gap, we utilized a comprehensive strategy that integrated bioinformatics evaluations of TCGA-ESCA datasets alongside immunohistochemistry and functional experiments conducted in ESCA cell lines. Furthermore, we constructed and validated a clinicopathological diagnostic model incorporating IGFBP1 expression along with clinical variables (age, gender, BMI, smoking status), which demonstrated favorable discriminatory ability and clinical net benefit for ESCA risk prediction. Beyond conventional functional enrichment analyses (GO/KEGG) and pathway activity profiling (GSEA), we systematically investigated IGFBP1’s DNA methylation and mutation status, and its correlation with m6A modification regulators and performed weighted gene co-expression network analysis (WGCNA) to identify key co-expressed gene modules associated with IGFBP1. *In vitro* functional experiments were conducted employing CCK-8, Transwell, flow cytometry assays and *in vivo* xenograft tumor models were conducted to confirm the participation of IGFBP1 in promoting the proliferation, migration and cell cycle progression of ESCA. These findings collectively nominate IGFBP1 as a novel biomarker for ESCA, offering both diagnostic utility and mechanistic insights for therapeutic targeting.

## Materials and methods

### Acquisition of data and processing

RNA sequencing (RNA-seq) datasets, along with clinical data, were sourced from the TCGA database (174 ESCA and 11 normal tissues). Following the exclusion of low-quality samples (n=3) that exhibited read counts below 10 million, the raw RNA-seq data were subsequently standardized to transcripts per million (TPM) utilizing the DESeq2 R package (version 1.38.3). Clinical variables including TNM staging (AJCC 8th edition), survival endpoints (OS, DSS, PFI), and histologic subtypes were harmonized using TCGA clinical files.

### Functional enrichment analysis

After 163 ESCA cases were stratified into two cohorts (IGFBP1-high and IGFBP1-low), the R package “Limma” was employed to detect differentially expressed genes (DEGs) among these two groups, utilizing a criterion of log2 fold change (FC) exceeding 1 and P < 0.05. Gene ontology (GO) profiling was conducted to classify DEGs through the “GOplot”. The “ClusterProfiler” was utilized for GSEA to uncover IGFBP1-related biological phenotypes.

### Immune infiltration analysis

In order to thoroughly characterize the IGFBP1-related immune landscape of ESCA, two synergistic methodologies were implemented for the analysis of immune infiltration. Initially, ssGSEA integrated with the GSVA R package was utilized to derive relative enrichment scores for various immune cell subsets, thereby reflecting their functional activities in individual tumor specimens. Simultaneously, the CIBERSORT method was applied to measure the proportional distribution of 22 immune cell subtypes (23). The correlations between IGFBP1 and the immune infiltration profiles obtained from both analytical methods were analyzed using Spearman’s test. Furthermore, the Wilcoxon test was implemented to assess variations in immune cell infiltration between the two cohorts, thereby facilitating a robust characterization of the ESCA immune microenvironment.

### Validation of the correlation between IGFBP1 expression with m6A regulators in ESCA

A meticulously compiled inventory of 21 established regulators of m6A modification has been assembled, which includes methyltransferases (METTL3, METTL16, METTL14, WTAP), demethylases (FTO, ALKBH5), as well as m6A-related genes (YTHDF1, YTHDF2, YTHDF3, and IGF2BP1, IGF2BP2, IGF2BP3) (24). The expression levels of these regulators within the TCGA-ESCA dataset were extracted, and their association with IGFBP1 expression was evaluated employing Spearman’s rank correlation coefficient.

### Assessment of the prognostic relevance of IGFBP1 in ESCA

The impact of IGFBP1 expression on OS, DSS, and PFI in ESCA patients was examined through Kaplan-Meier method. To ascertain the independent prognostic implication of IGFBP1 in ESCA, univariate and multivariate Cox analyses were conducted. Variables exhibiting P < 0.1 in the univariate analysis were used for the multivariate Cox regression model to account for potential confounding variables, including age, sex, TNM staging, and histological subtype. ROC curves were employed to examine the discriminative potential of IGFBP1 in ESCA. Multivariate Cox regression was executed to explore the interrelationships among variables within the prognostic model. The analysis of clinicopathological characteristics and risk scores was conducted utilizing the regplot, survival, and rms packages.

### Single cell analysis of IGFBP1 in ESCA

The scRNA-seq data pertaining to ESCA was obtained from the GEO database under the accession number GSE196756 (25). The Seurat package (version 4.3.0) facilitated the processing of the scRNA-seq data, which encompassed several steps such as quality control—where cells exhibiting less than 200 genes or over 20% mitochondrial RNA content were excluded—normalization, dimensionality reduction through Principal Component Analysis and subsequent cell type annotation using established canonical marker genes. The expression levels of IGFBP1 across distinct cell types were depicted through UMAP visualizations, and expression distribution was further quantified via violin plots. Cell-cell interaction analysis was performed to evaluate the interaction number and interaction strength across all annotated cell types. Gene Set Variation Analysis (GSVA) was conducted on the single-cell transcriptomic profiles using R software. In silico knockdown of IGFBP1 was implemented using R packages, followed by GSVA enrichment analysis, and comparative analysis of the GSVA results before and after IGFBP1 knockdown was performed to identify the affected biological processes.

### *In vitro* validation of IGFBP1

In order to explore the functional significance of IGFBP1 in ESCA, *in vitro* experiments were performed utilizing two cell lines, KYSE-150 and KYSE-30. IGFBP1 expression was silenced using small interfering RNA (siRNA) specific to IGFBP1. Small interfering RNAs (siRNAs) targeting IGFBP1 (si-IGFBP1-1: 5`-UUCCAUUUUUUGAUGUUGUG-3`; si-IGFBP1-2: 5`-AAAAUUUGGAAAUUUCUUCUC-3`; si-IGFBP1-3: 5`-AUAAAAUCCAUUCUUGUUGCA-3`; were obtained from GenePharma (Shanghai, China). Cell proliferation was examined utilizing the Cell Counting Kit-8 (CCK-8) assay. For the assessment of the migratory abilities of the cells, transwell migration and invasion experiments were conducted, employing matrigel-coated chambers specifically for the invasion assessment.

### RT-qPCR and Western blot Analysis

Total RNA was extracted from cells using TRIzol reagent (Invitrogen), and the synthesis of cDNA was conducted utilizing the PrimeScript RT Reagent Kit (TaKaRa, Japan). The relative expression of IGFBP1 quantified via the 2^-ΔΔCt^ algorithm, with GAPDH as reference gene. Sequences of the primers were: IGFBP1 forward: 5’-GGCTCTCCATGTCACCAACA-3’, reverse: 5’-CCATTCCAAGGGTAGACGCA-3’; GAPDH forward: 5’-AATGACCCCTTCATTGAC-3’, reverse: 5’-TCCACGACGTACTCAGCGC-3’. For Western blot assays, total protein was extracted via lysis buffer with inhibitors of protease and phosphatase (Roche, Switzerland). Concentrations of the extracted proteins were quantified via the BCA Protein Assay Kit. Following normalization of protein concentrations, equal quantities of protein were resolved by 10% SDS-PAGE, with separated proteins were then transferred to PVDF membranes. The PVDF membranes underwent blocking using 5% skim milk for 1h, then exposed to primary antibodies against IGFBP1 (1:1000, Abcam) and GAPDH (1:5000) overnight at 4 °C. After washing with TBST, membranes were incubated with horseradish peroxidase (HRP)-conjugated secondary antibodies (1:5000, Cell Signaling Technology) for 1 hour at room temperature. Protein bands were visualized via ECL Detection Kit, and band intensities were determined using ImageJ software (v1.8.0).

### *In vivo* tumor formation assay

Male BALB/c nude mice (18–22 g) were purchased from Zhi Yuan Bio-technology Co., Ltd (Guangzhou), and housed in specific pathogen-free environment. Stable IGFBP1-knockdown KYSE-30 and KYSE-150 cell lines were constructed via lentiviral transduction. Briefly, sh-IGFBP1 (target sequence: 5′-CCACCATTCACATTTGATGTA-3′) or sh-NC together with packaging plasmids were co-transfected into 293T cells to produce lentivirus. KYSE-150 and KYSE-30 cells were infected with harvested viral supernatants and subjected to 4 μg/mL puromycin screening to establish stable cell lines. For subcutaneous xenograft experiments, the validated stable cells were resuspended in sterile PBS at a density of 5×10^6^ cells/100 μL for inoculation. A volume of 100μl of this cell suspension was injected into the right flank region of individual BALB/c nude mice, with a sample size of 6 mice per group to ensure statistical power. Tumor size was measured every 3 days using a vernier caliper, and computed using V = (length × width²)/2 formula. Euthanasia was performed by gradual CO_2_ inhalation at a flow rate of 50% chamber volume per minute to minimize animal distress; following loss of consciousness and respiratory arrest, cervical dislocation was conducted as a secondary method to confirm death and ensure consistent humane endpoints. All animal experimental protocols were reviewed and approved by the Animal Ethics Committee of School of Medicine, Kashi Universality (number: KSU-AEC-A-2025-002), and all procedures were carried out in strict accordance with national and international guidelines for the care and use of laboratory animals.

### Statistical analysis

Statistical analyses were conducted via R software (v4.3.1) and GraphPad Prism (v9.0). Comparisons between two groups were performed using the Student’s t-test (normal distribution) or Wilcoxon rank-sum test. Categorical variables were compared using Pearson’s chi-square test, Yates’ correction test, or Fisher’s exact test according to expected frequencies. Correlations were analyzed using Spearman’s rank correlation coefficient. Survival analysis was conducted via the Kaplan-Meier method with log-rank test and Cox proportional hazard analysis.

## Results

### IGFBP1 is significantly overexpressed in ESCA

We conducted an assessment of IGFBP1 expression in ESCA utilizing TCGA data. Notably, IGFBP1 was upregulated in ESCA relative to normal tissues (**Figure 1**). This observation was corroborated through the analysis of matched ESCA tissue specimens (**Figure 1**). Additionally, the receiver operating characteristic (ROC) analysis indicated that IGFBP1 possesses moderate diagnostic value (AUC = 0.688) (**Figure 1**). The IHC analysis on ESCA tissue microarray indicated strong expression of IGFBP1 in tumor tissues (**Figure 1D**; **Supplementary Figure 1A**). Furthermore, we observed distinct subcellular localization of IGFBP1 in ESCA cells: the expression of IGFBP1 was very low in the cell nucleus, whereas its expression was significantly higher in the cell membrane and cytoplasm (**Supplementary Figure 1C**). Taken together, these data reveal that IGFBP1 may represent a novel and promising biomarker for facilitating early and accurate clinical diagnostic assessments in ESCA.

**Figure 1:**
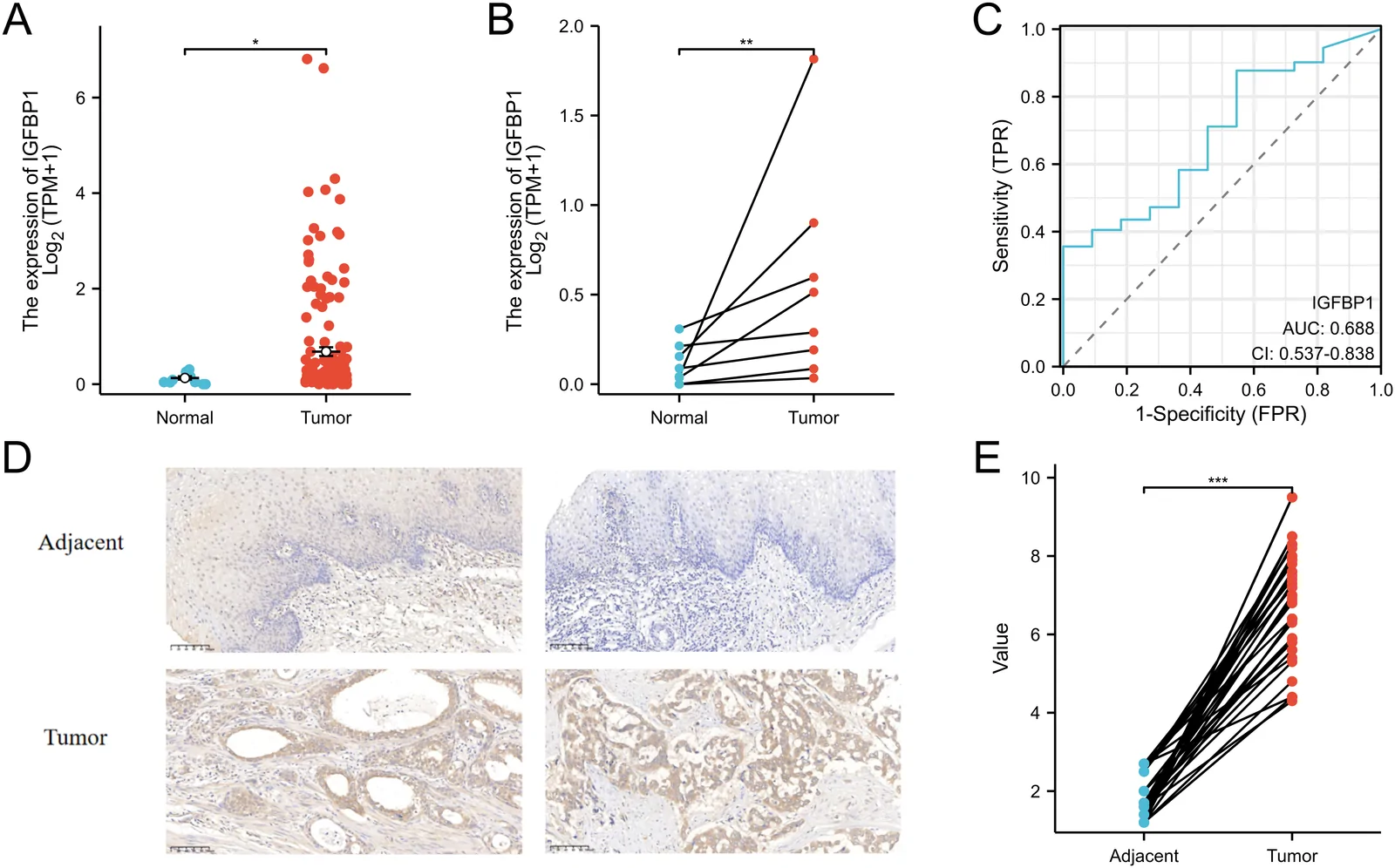
IGFBP1 is significantly overexpressed in ESCA. **(A)** IGFBP1 expression in TCGA-ESCA. **(B)** IGFBP1 expression in TCGA-ESCA (matched tissues). **(C)** ROC value of IGFBP1 in ESCA. **(D)** IGFBP1 expression in ESCA tissues. **(E)** Validation of IGFBP1.

### IGFBP1 expression is closely associated with ESCA clinical features

We examined the associations between IGFBP1 expression and key clinical parameters in patients diagnosed with ESCA. Our findings revealed notable correlations between IGFBP1 expression and multiple clinical parameters (**Table 1**). In particular, we noted associations with various clinical factors, including the clinical M stage, overall clinical stage, pathological stage, patient weight, body mass index (BMI), histological classification, histological grading, history of reflux, tumor location, and the administration of radiation therapy (**Figure 2A**). By contrast, there were no direct correlations between IGFBP1 expression and patient age or gender (**Figure 2K**). The logistic regression analysis provided additional evidence that the expression levels of IGFBP1 serve as an independent prognostic factor for ESCA, after adjusting for other confounding clinical variables (**Table 1**). In particular, elevated expression of IGFBP1 was markedly correlated with clinical T stage, patient body weight, histological classification, tumor location, and the administration of radiation therapy. These findings highlight the clinical relevance of IGFBP1 in ESCA and its potential implications for patient management.

**Table 1:** The clinicopathological features of ESCA patients.

Characteristics	IGFBP1-Low	IGFBP1-High	P
n	80	83	
Age, n (%)			0.242^a^
<= 60	37 (22.7%)	46 (28.2%)	
> 60	43 (26.4%)	37 (22.7%)	
Gender, n (%)			0.441^a^
Female	13 (8%)	10 (6.1%)	
Male	67 (41.1%)	73 (44.8%)	
Clinical T stage, n (%)			0.084^b^
T1	6 (11.5%)	0 (0%)	
T2	7 (13.5%)	3 (5.8%)	
T3	15 (28.8%)	17 (32.7%)	
T4	2 (3.8%)	2 (3.8%)	
Clinical M stage, n (%)			0.046^a*^
M0	25 (48.1%)	16 (30.8%)	
M1	3 (5.8%)	8 (15.4%)	
Primary therapy outcome, n (%)			0.576^b^
PD	5 (5.3%)	5 (5.3%)	
SD	5 (5.3%)	2 (2.1%)	
PR	1 (1.1%)	2 (2.1%)	
CR	47 (50%)	27 (28.7%)	
Weight, n (%)			< 0.001^a*^
<= 70	48 (29.8%)	28 (17.4%)	
> 70	31 (19.3%)	54 (33.5%)	
Height, n (%)			0.460^a^
< 170	25 (16.2%)	22 (14.3%)	
>= 170	50 (32.5%)	57 (37%)	
Histological type, n (%)			< 0.001^a*^
Adenocarcinoma	24 (14.7%)	57 (35%)	
Squamous cell carcinoma	56 (34.4%)	26 (16%)	
Residual tumor, n (%)			0.702^b^
R0	61 (45.5%)	60 (44.8%)	
R1	7 (5.2%)	4 (3%)	
R2	1 (0.7%)	1 (0.7%)	
Alcohol history, n (%)			0.266^a^
No	20 (12.5%)	27 (16.9%)	
Yes	59 (36.9%)	54 (33.8%)	
Tumor location, n (%)			0.025^b*^
Distal	48 (29.6%)	66 (40.7%)	
Mid	28 (17.3%)	14 (8.6%)	
Proximal	3 (1.9%)	3 (1.9%)	
Radiation therapy, n (%)			0.003^a*^
No	48 (33.1%)	60 (41.4%)	
Yes	27 (18.6%)	10 (6.9%)	

^a^Represents Pearson`s chi-square test. ^b^Represents Yates` correction test. *Statistically significant difference (p< 0.05). Bold value indicate significant difference (p<0.05).

**Figure 2:**
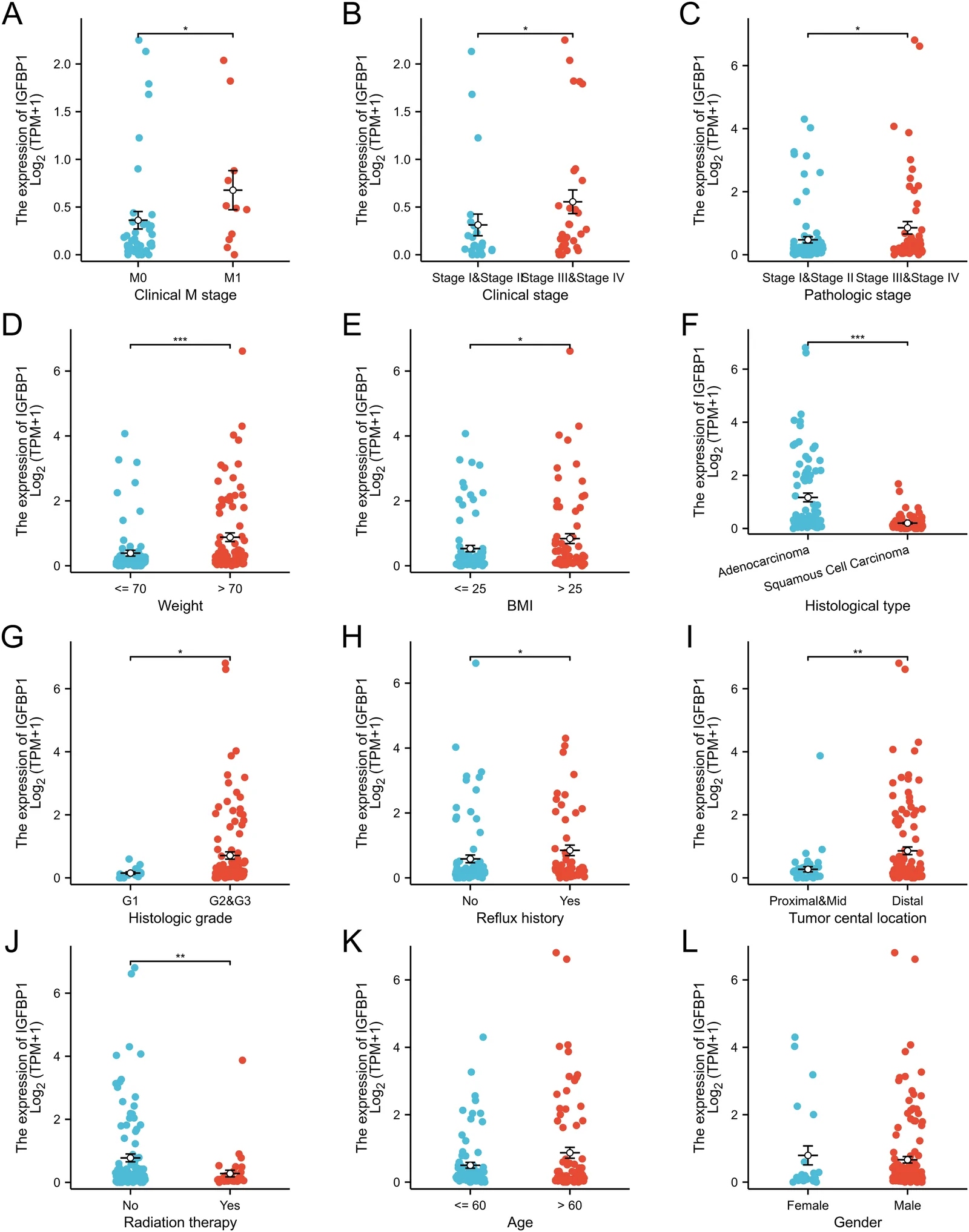
Association of IGFBP1 expression with clinical variables in ESCA patients. **(A)** Clinical M stage. **(B)** Clinical stage. **(C)** Pathological stage. **(D)** Weight. **(E)** BMI. **(F)** Histological type. **(G)** Histological grade. **(H)** Reflux history. **(I)** Tumor location. **(J)** Radiation therapy. **(K)** Age. **(L)** Gender.

### Curation and validation of IGFBP1-related diagnostic model in ESCA

After matching transcriptomic profiles and clinical annotations, a total of TCGA-ESCA samples comprising primary ESCA and matched normal adjacent tissues were included for analysis. Clinical variables were selected based on data completeness and clinical relevance, including age at diagnosis, gender, BMI, smoking status (current/former vs never smoker), and IGFBP1 expression. A nomogram was developed to translate regression coefficients into a clinically accessible risk prediction tool, assigning points to each predictor (age, gender, BMI, smoking status, IGFBP1 expression) and mapping total points to predicted probabilities of ESCA with a calibration threshold set at 0.9 (**Figure 3**). A forest plot was generated to quantify the independent effect of each variable, revealing that log-transformed IGFBP1 expression was an independent predictor of ESCA status (OR = 1.791, P = 0.025), while no other clinical variables (age, gender, BMI, smoking status) reached statistical significance in this cohort (P > 0.05) (**Figure 3**). TCGA-ESCA Data were partitioned at the patient level into a training set (70%) and a hold-out validation set (30%) to avoid information leakage by ensuring no overlap of tumor/normal pairs between sets. In the training cohort, the model achieved an AUC of 0.884 (95% CI: 0.806–0.961) indicating strong discriminatory ability, and calibration analysis showed excellent agreement between predicted and observed probabilities with the calibration curve closely aligning with the ideal 45° reference line; decision curve analysis (DCA) demonstrated that the model provided a net clinical benefit across a wide range of risk thresholds (0.2–0.8), superior to the “treat all” or “treat none” strategies (**Figure 3C**). Bootstrap resampling confirmed robust internal validity, with the bias-corrected calibration curve remaining close to the ideal line and confirming minimal overfitting. In the independent hold-out set, the model maintained an AUC of 0.738 (95% CI: 0.402–1.000), demonstrating reproducible discriminatory power, and DCA in the hold-out set further validated the model’s clinical utility across relevant risk thresholds (**Figure 3F**). A multilayer perceptron (MLP) with an input layer matching the logistic regression predictors (age, gender, BMI, smoking, IGFBP1) was trained for comparative purposes, achieving an AUC of 0.835 (95% CI: 0.619–1.000) in the training set. This performance was comparable to the logistic regression model (**Figure 3I**). Collectively, these findings indicate that log-transformed IGFBP1 expression is an independent predictor of ESCA status in the TCGA cohort, the multivariate logistic regression model exhibits strong discriminatory ability (training AUC = 0.884) and reproducible performance.

**Figure 3:**
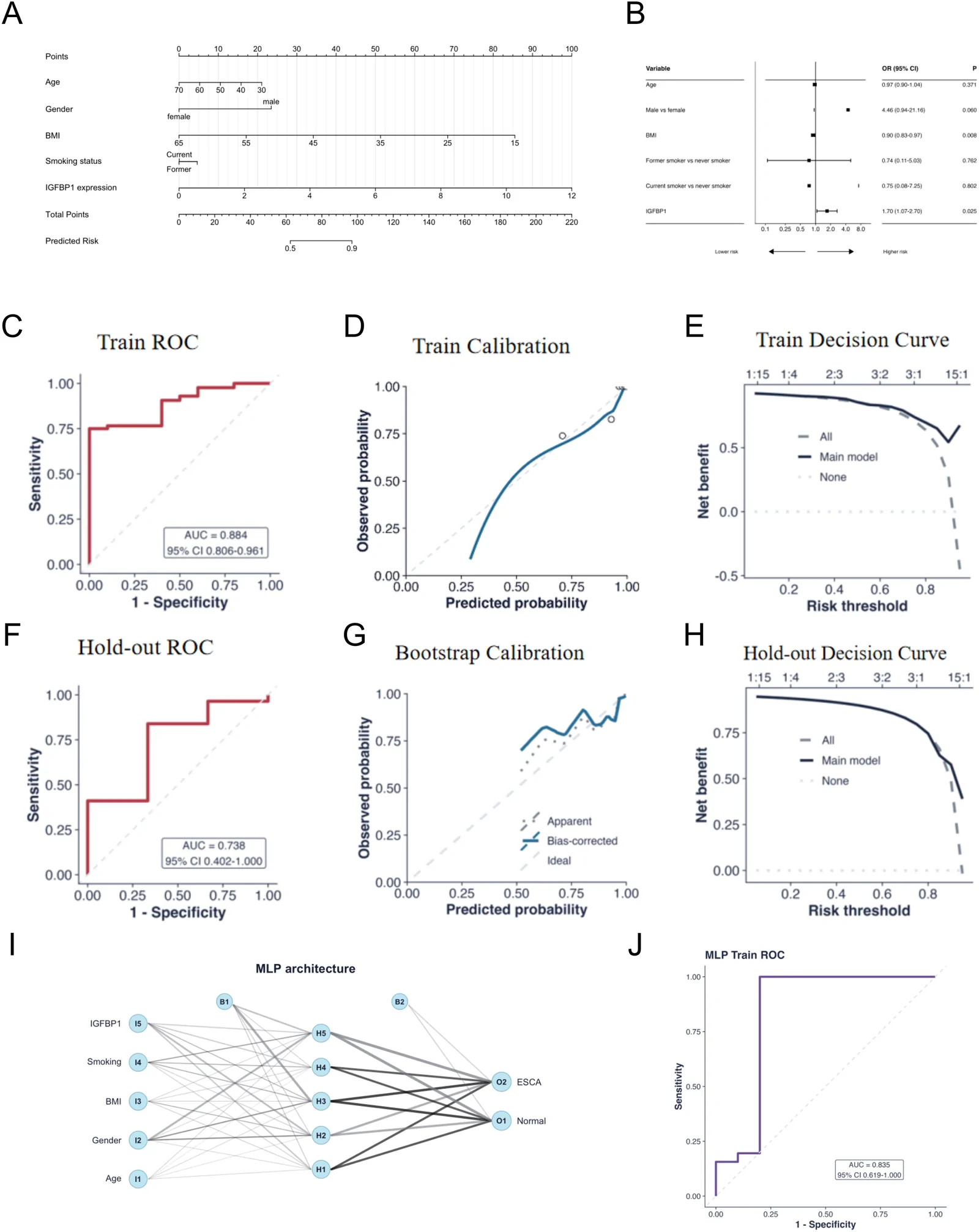
Development and validation of an IGFBP1-based clinicopathological diagnostic model for ESCA. **(A)** Nomogram integrating age, gender, BMI, smoking status. **(B)** Forest plot of univariate logistic regression analysis. **(C)** ROC curve of the diagnostic model. **(D)** Calibration curve of the diagnostic model. **(E)** DCA of the diagnostic model. **(F)** ROC curve of the diagnostic model in the hold-out validation cohort. **(G)** Bootstrap-calibrated calibration curve of the diagnostic model. **(H)** DCA of the diagnostic model in the hold-out validation cohort. **(I)** MLP model, featuring input nodes for clinical variables and IGFBP1 expression. **(J)** ROC curve of the MLP model in the training cohort.

### WGCNA analysis of IGFBP1 in ESCA

Weighted gene co-expression network analysis (WGCNA) was performed on the gene expression profiles of TCGA-ESCA samples, and the gene dendrogram with module color assignment was generated (**Figure 4**). Notably, genes closely related to IGFBP1 were clustered into the green module. A total of 2823 genes were clustered into distinct co-expression modules, and module-trait correlation analysis was performed to identify modules associated with control and treatment phenotypes (**Figure 4**). To construct a scale-free network, soft threshold powers were evaluated, with scale independence and mean connectivity assessed to determine the optimal threshold (**Figure 4C**). Subsequently, gene set variation analysis (GSVA) was conducted on the genes within the IGFBP1-related co-expression module, revealing that the KRAS signaling pathway was one the most significantly affected biological processes (**Figure 4**), suggesting that IGFBP1 may regulate ESCA progression potentially through modulating the KRAS signaling pathway.

**Figure 4:**
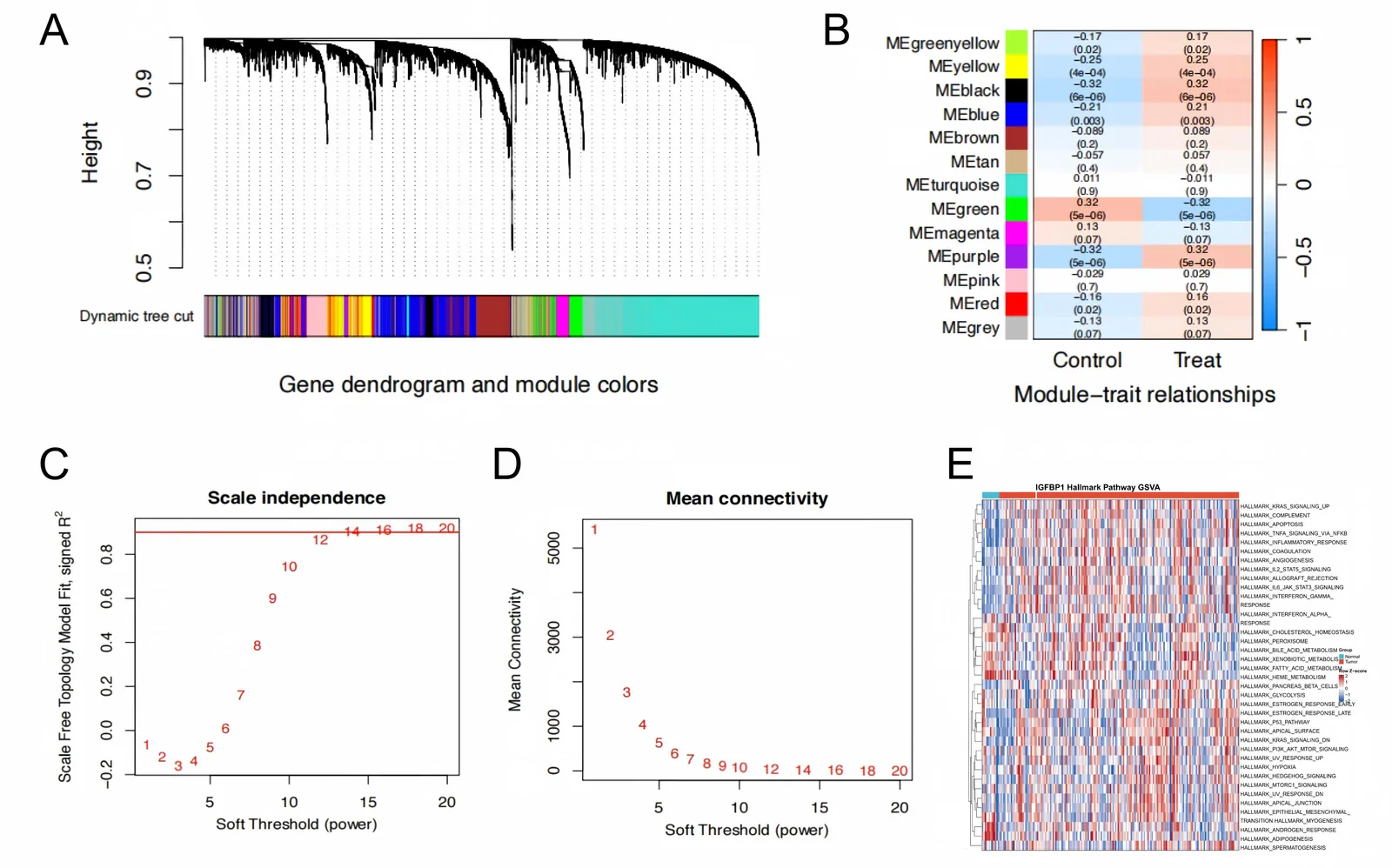
WGCNA and functional analysis of IGFBP1-related genes in ESCA. **(A)** Gene dendrogram and co-expression module color assignment. **(B)** Module-trait relationship heatmap. **(C)** Scale independence plot for soft threshold selection. **(D)** Mean connectivity plot for network topology validation. **(E)** GSVA heatmap of IGFBP1-related module genes.

### Identification of IGFBP1-related DEGs associated with IGFBP1 in ESCA

To further explore the IGFBP1-related molecular mechanisms in ESCA, we performed differential expression analysis. Gene expression profiles were contrasted between ESCA cases and normal cases. The Wilcoxon test was applied to identify genes with significant expression changes, using a threshold of padj < 0.05 and |log2 FC| > 1. A total of 1,247 DEGs, including 684 upregulated and 563 downregulated genes were selected (**Figure 5**). The top 10 genes most significantly correlated with IGFBP1 were FGG, APOA2, ORM1, GC, CLCA1, DEFA6, APOB, APOC3, DEFA5 and FGB (**Figure 5**). We conducted enrichment analysis to decipher the functional significance of the DEGs. GO enrichment highlighted significant terms in biological processes (e.g., “protein-lipid complex remodeling,” “assembly of protein-lipid complex”), cellular components (“blood microparticle”), and molecular functions (“receptor ligand activity”). KEGG pathway analysis identified “neuroactive ligand-receptor interaction,” “fat digestion and absorption,” and “PPAR signaling pathway” (**Figure 5**). GSEA was employed to evaluate the enrichment of DEGs in the context of IGFBP1 expression. GSEA identified significant enrichment in pathways such as “remodeling of protein containing complex”, “assembly of protein-lipid complex”, and “triglyceride rich plasma lipoprotein particle” (**Figure 5**). These results indicate that IGFBP1-associated DEGs are involved in critical tumorigenic processes, which may suggest that IGFBP1 could potentially participate in the regulation of these oncogenic pathways in ESCA.

**Figure 5:**
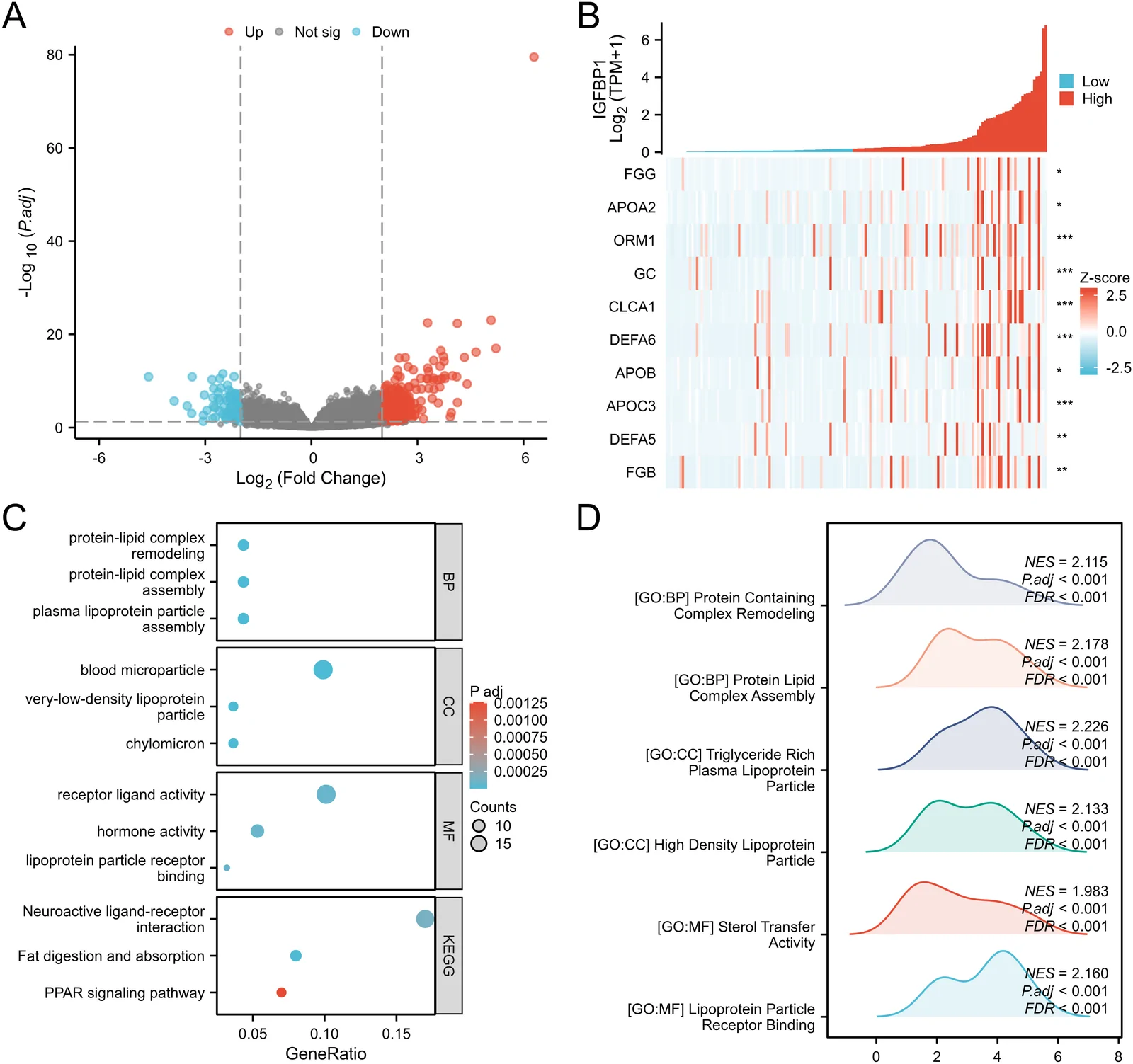
DEGs and functional analysis related to IGFBP1 in ESCA. **(A)** Identification of 1,247 DEGs. **(B)** Top 10 DEGs presented in a heatmap. **(C)** GO/KEGG enrichment analysis of IGFBP1-associated DEGs. **(D)** GSEA analysis showing enrichment in cancer-related pathways.

### Single-cell resolution analysis of IGFBP1 in ESCA

To dissect the cellular and molecular context of IGFBP1 in ESCA, we performed single-cell clustering and annotation on the GSE196756, classifying cells into distinct types including common myeloid progenitors, T cells, smooth muscle cells, monocytes, epithelial cells, neutrophils, B cells, tissue stem cells, neurons and endothelial cells (**Figure 6**). This established a comprehensive cellular landscape of ESCA, providing a framework for subsequent IGFBP1-focused analyses. To map IGFBP1 expression, we used UMAP and quantified expression distribution via violin plots (**Figure 6B**). IGFBP1 was predominantly expressed in smooth muscle cells, with near-undetectable expression in all other annotated cell types, indicating dataset-specific, cell type restricted expression of IGFBP1 in ESCA. We next characterized intercellular interactions and pathway-specific communications. The number of interactions and interaction strength illustrated the complex cell–cell interaction networks across all annotated cell types, with CMP, T cells, B cells, and monocytes exhibiting extensive interactions (**Figure 6D**). To further explore the functional implications of IGFBP1 in ESCA, we performed GSVA enrichment analysis on the single-cell transcriptomic profiles (**Figure 6**), followed by in silico knockdown of IGFBP1 and subsequent GSVA analysis (**Figure 6**). Comparative analysis of the GSVA results before and after IGFBP1 knockdown revealed distinct pathway enrichment signatures, with the IGF signaling pathway and cell migration adhesion were identified as a key affected biological processes (**Figure 6**). To further validate this finding, we performed single-cell expression analysis of core genes in the IGF family, including IGF1, IGF2, IGF1R, and IGF2R. UMAP visualization (**Supplementary Figure 2A**) and violin plot quantification demonstrated the cell type-specific expression patterns of these IGF family members across the ESCA cellular landscape (**Supplementary Figure 2E**). Subsequent correlation analysis revealed no significant linear correlation between IGFBP1 expression and the expression of IGF1, IGF1R, or IGF2R, while only a weak positive correlation was observed between IGFBP1 and IGF2 (**Supplementary Figure 2I**), further clarifying the expression relationship between IGFBP1 and other key components of the IGF signaling axis in ESCA.

**Figure 6:**
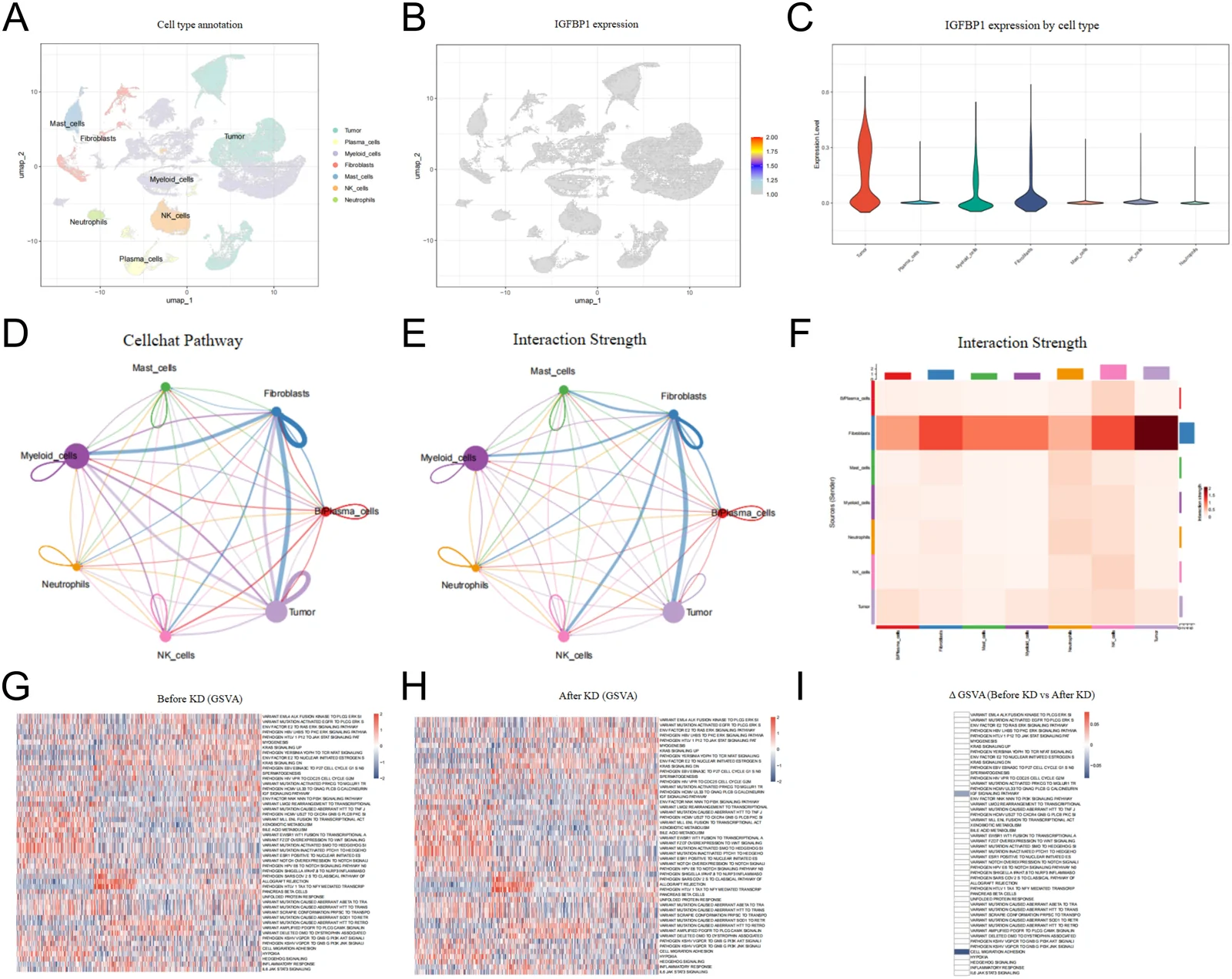
Single cell analysis of IGFBP1 in ESCA.**(A)** Cell type annotation in GSE196756. **(B)** UMAP of IGFBP1 expression distribution, with a color gradient (0.0–0.6) indicating expression level. **(C)** Violin plot of IGFBP1 expression across cell types. **(D)** Network plot of intercellular interaction numbers. **(E)** Network plot of interaction strength. **(F)** Validation of network interaction strength. **(G)** GSVA analysis before virtual knockout. **(H)** GSVA analysis after virtual knockout. **(I)** Comparison of GSVA before KD and after KD.

### Single-cell transcriptomic profiling of cell migration adhesion related gene signatures in ESCA

To further validate the link between IGFBP1 and cell migration adhesion, we analyzed the single-cell expression patterns and correlation coefficients of core cell migration signature genes, including ITGA5, MMP2, MMP9, VIM, CDH1, and CDH2. UMAP visualization (**Figure 7A**) and violin plot quantification demonstrated cell type-specific enrichment of these cell migration adhesion factors in SMCs and adjacent cell populations, mirroring the spatial expression pattern of IGFBP1 (**Figure 7G**). Correlation analysis revealed a strong positive association between IGFBP1 expression and the markers ITGA5, MMP2 and VIM, while no significant linear correlation was observed between IGFBP1 and MMP9 (p = 0.444), CDH1 (p = 0.621), and CDH2 (p = 0.5493) (**Figure 7M**). These results suggest that IGFBP1 may modulate cell migration in ESCA potentially through regulating mesenchymal transition-related gene expression, and ITGA5, MMP2 and VIM may act as key downstream effectors mediating its pro-migration function.

**Figure 7:**
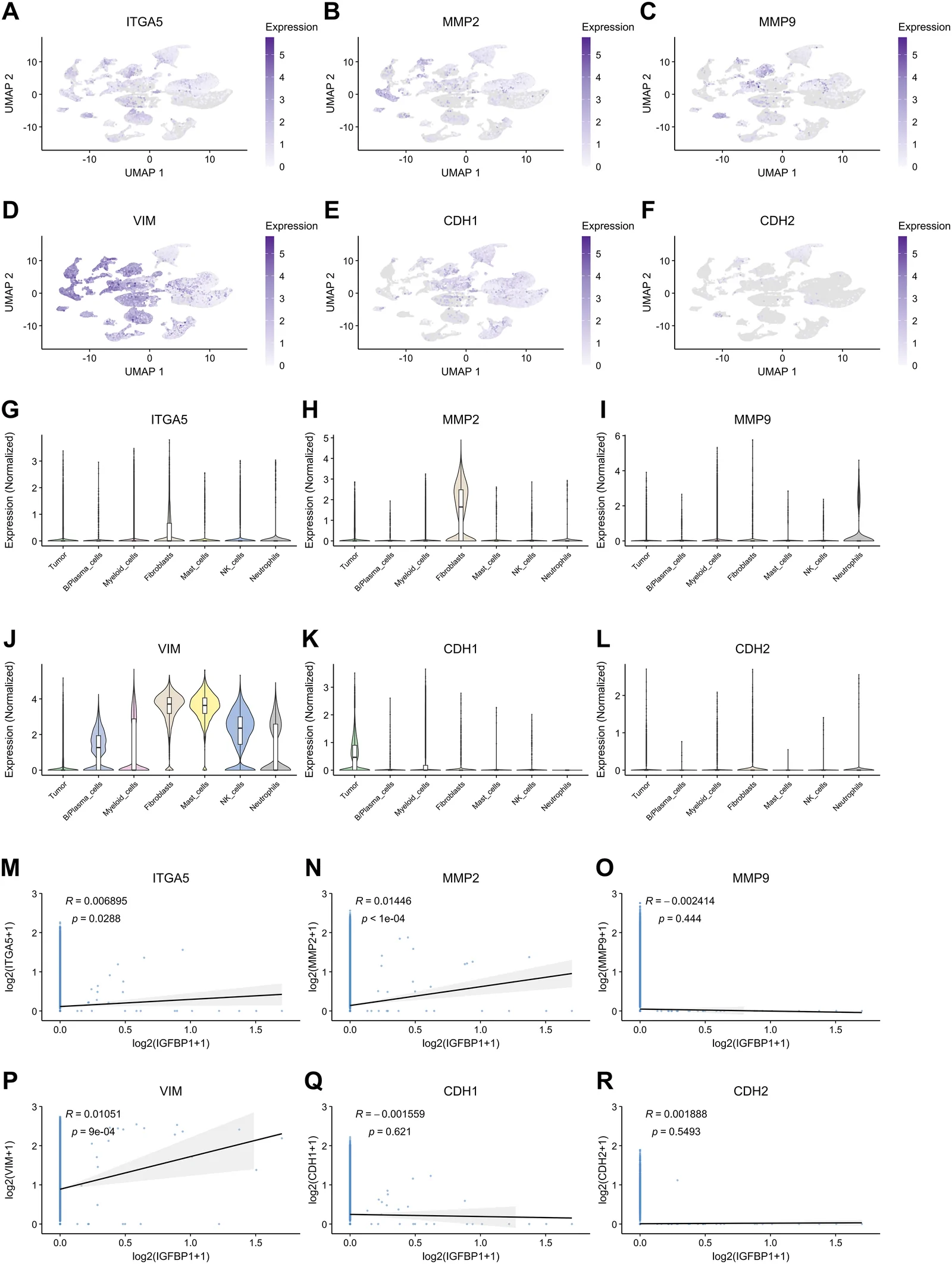
IGFBP1 correlates with core cell migration markers in ESCA at single-cell resolution. UMAP visualization of the expression of EMT-related genes **(A)** ITGA5, **(B)** MMP2, **(C)** MMP9, **(D)** VIM, **(E)** CDH1, **(F)** CDH2. Violin plots quantifying the normalized expression levels of **(G)** ITGA5, **(H)** MMP2, **(I)** MMP9, **(J)** VIM, **(K)** CDH1, **(L)** CDH2. Scatter plots showing the correlation between IGFBP1 expression and the expression of **(M)** ITGA5, **(N)** MMP2, **(O)** MMP9, **(P)** VIM, **(Q)** CDH1, **(R)** CDH2.

### Immune infiltration analysis using ssGSEA and correlation with IGFBP1 expression

To examine IGFBP1-related immune signature in ESCA tumor microenvironment, we performed ssGSEA/CIBERSORT method. The analysis quantified the enrichment scores of 24 immune cell subsets according to the cell marker gene list (**Figure 8**). Significant correlations were observed with decreased infiltration of Tcm cells, NK56dim cells, and total NK cells (**Figure 8B**). Additionally, Wilcoxon tests comparing high and low IGFBP1 expression groups revealed altered immune profiles, including reductions in Tcm cells, NK56dim cells and NK cells (**Figure 8E**). Conversely, IGFBP1 was positively correlated with pro-tumor/suppressive immune subtypes: M2 macrophage, Th17 cells and pDC cells infiltration (**Supplementary Figure 3A**). To validate these trends, we performed CIBERSORT analysis (**Supplementary Figure 4**). Specifically, a robust inverse association was observed between IGFBP1 values and the abundance of resting macrophages M1, dendritic cells resting, and mast cells resting, all of which are critical for initiating and sustaining anti-tumor immune surveillance (**Supplementary Figure 4B**). These results align with the ssGSEA data, collectively indicating that IGFBP1 may reshape the tumor immune microenvironment toward an immunosuppressive phenotype, characterized by reduced infiltration of pro-inflammatory/anti-tumor immune cells and increased accumulation of pro-tumorigenic immune subsets.

**Figure 8:**
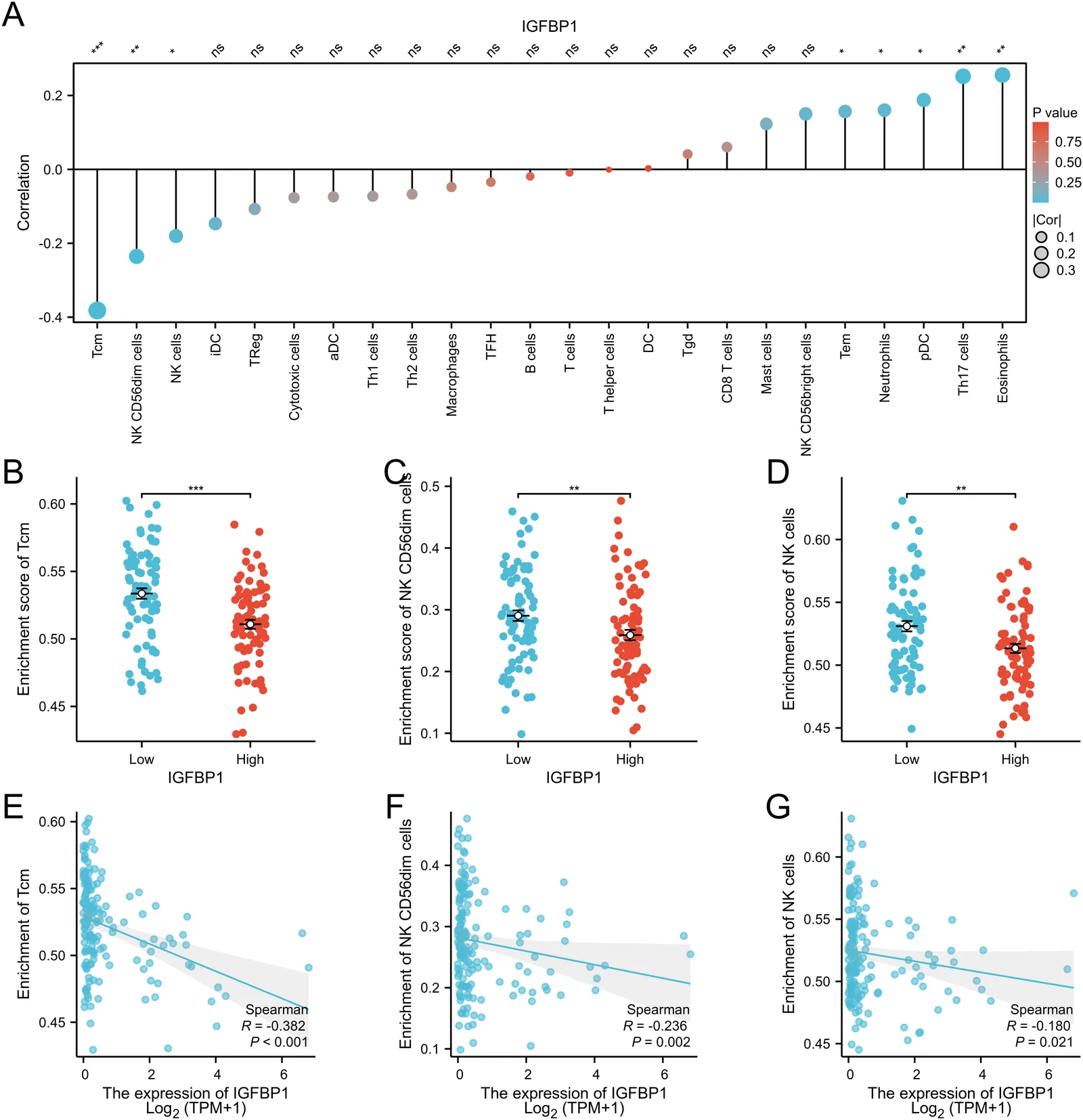
IGFBP1-related immune signature in ESCA. **(A)** The examination of IGFBP1-related immune cell subtypes in ESCA. **(B)** Tcm cells. **(C)** NK CD56dim cells. **(D)** NK cells. **(E)** Tcm cells. **(F)** NK CD56dim cells. **(G)** NK cells.

### Correlation of IGFBP1 expression with m6A regulators in ESCA

To further explore the molecular regulatory mechanisms underlying the aberrant high expression of IGFBP1 in ESCA, we investigated the promoter methylation profile of IGFBP1 in ESCA. We identified 12 differentially methylated CpG sites in the promoter region of IGFBP1 in ESCA (**Supplementary Figure 5**). The promoter methylation levels of IGFBP1 was up regulated in ESCA according to our analysis using UALCAN database (**Supplementary Figure 5**). Furthermore, survival analysis demonstrated that ESCA patients with high methylation levels of IGFBP1 promoter CpG sites (cg00110785, cg05660795) exhibited significantly better overall survival than those with low methylation levels (**Supplementary Figure 5C**). To further investigate the genetic alterations of IGFBP1, we accessed the cBioPortal database, analyzing eight datasets comprising 1492 ESCA samples. Genetic alterations in the IGFBP1 gene were identified in 3% of ESCA patients (**Supplementary Figure 6**). Survival analysis revealed that ESCA patients with IGFBP1 genetic alterations had significantly worse OS, and DSS, while no significant difference was observed in disease-free survival (DFS) and progression-free survival (PFS) (**Supplementary Figure 6B**). Subsequently, The association between IGFBP1 expression and the expression of 21 m6A regulator genes in TCGA-ESCA cohort was examined (**Figure 9**). Our analysis revealed that IGFBP1 exhibited a positive correlation with two m6A-associated genes in ESCA: IGF2BP1, IGF2BP3, and showed negative correlations with six m6A-related genes: ALKBH5, YTHDF2, FTO, METTL3, METTL14 and YTHDC1. The association between IGFBP1 and these m6A-related genes is visually represented in the scatter plots (**Figure 9B**). These results reveal a potential association between IGFBP1 expression and the expression profile of m6A regulatory genes in ESCA, suggesting that IGFBP1 may participate in m6A-mediated post-transcriptional regulation in ESCA.

**Figure 9:**
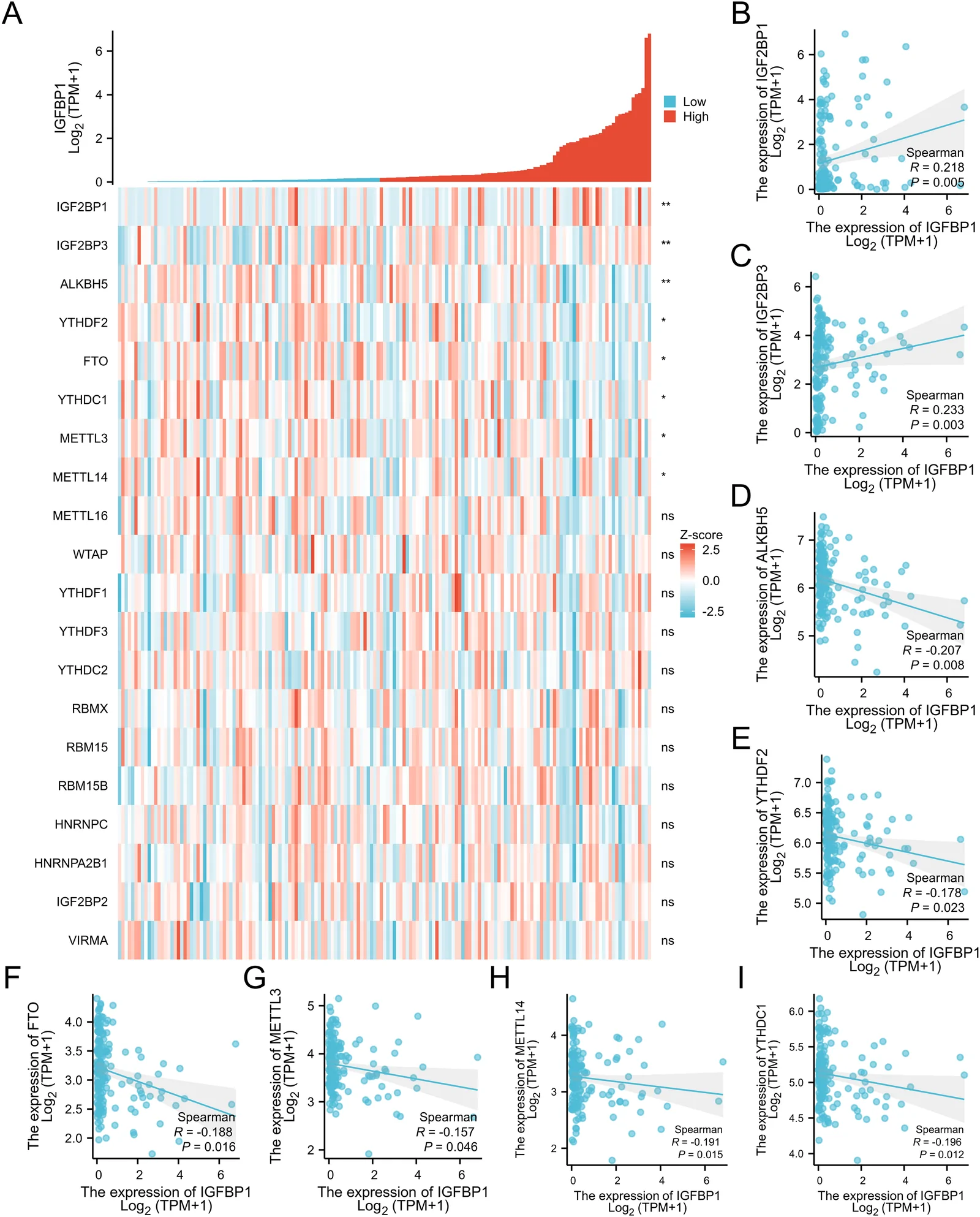
The correlations between IGFBP1 expression and m6A related genes in ESCA. **(A)** Heatmap illustration of the correlation between IGFBP1 and m6A-related genes. **(B)** IGF2BP1. **(C)** IGF2BP3. **(D)** ALKBH5. **(E)** YTHDF2. **(F)** FTO. **(G)** METTL3. **(H)** METTL14. **(I)** YTHDC1.

### IGFBP1 expression is associated with unfavorable outcomes in ESCA patients

We applied the Kaplan-Meier method to examine the correlation between IGFBP1.

expression(high/low groups) and OS, DSS, and PFI in ESCA. A significant.

correlation was observed between IGFBP1 expression and OS and DSS (p < 0.001;

**Figure 10A**), whereas no significant correlation was detected for PFI (p =0.251, **Figure 10**). We further stratified ESCA patients by clinical features to validate IGFBP1’s prognostic value. High IGFBP1 expression remained significantly linked to poor OS in multiple subgroups, including patients aged >60, male patients, those with adenocarcinoma histology, advanced clinical T stage (T3/T4), weight >70kg, BMI >25, smokers, patients without alcohol history, and those not receiving radiation therapy (**Supplementary Figure 5A**). These findings support IGFBP1 as a candidate prognostic biomarker linked to unfavorable survival outcomes in ESCA, particularly across key clinical patient subgroups.

**Figure 10:**
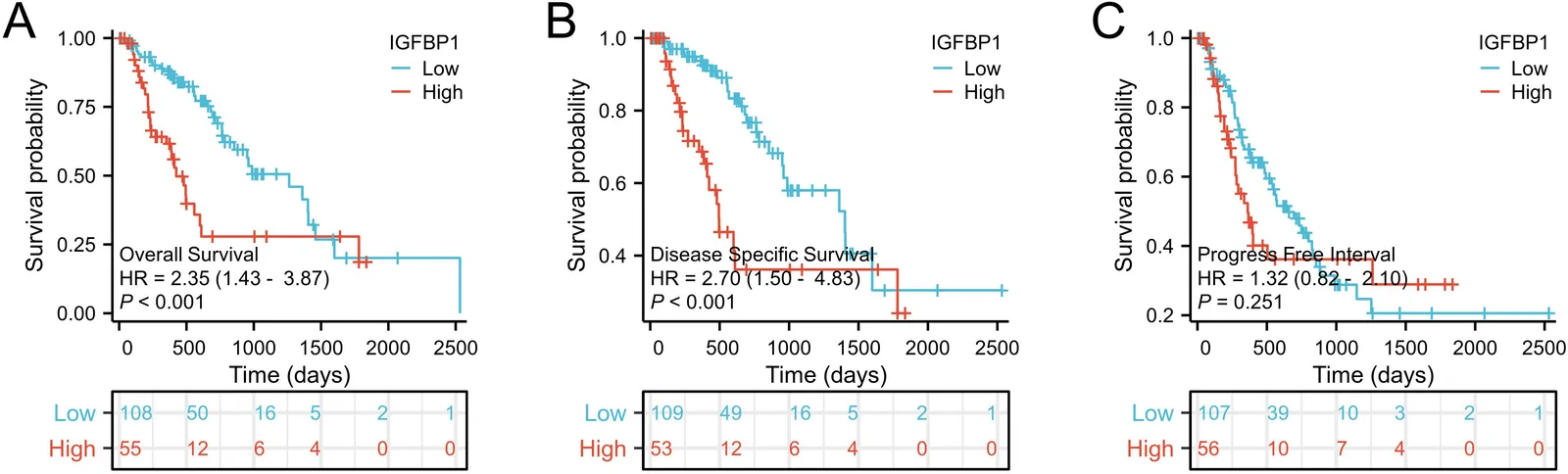
Kaplan-Meier curves illustrating the correlation between IGFBP1 expression levels and survival outcomes in ESCA patients. **(A)** OS. **(B)** DSS. **(C)** PFI.

### *In vitro* validation of IGFBP1 in ESCA

To dissect the biological relevance of IGFBP1 in ESCA, we assessed its expression in ESCA cell lines (KYSE150, KYSE30) relative to normal esophageal epithelial control (Het-1A). Our qRT-PCR and Western blot results demonstrated a statistically significant upregulation of IGFBP1 at both mRNA and protein levels in KYSE150 and KYSE30 cells relative to Het-1A (**Figure 11A**). We then performed IGFBP1 knockdown in KYSE150 and KYSE30 cells using specific siRNAs. Our qRT-PCR and Western blot analyses demonstrated that si-IGFBP1#1 and si-IGFBP1#2 effectively reduced IGFBP1 expression in ESCA cell lines (**Figure 11C**). Next, CCK-8 results indicated that silencing IGFBP1 significantly reduced KYSE150 and KYSE30 proliferation over 72h, versus the si-NC (**Figure 11G**). Transwell assays demonstrated that silencing IGFBP1 impaired the migratory and invasive abilities of both cell lines (**Figure 12A**). Our cell-cycle analysis showed that IGFBP1 silencing elevated the cell proportion arrested in G2/M phase (by 30%) and decreased S-phase entry, relative to si-NC (p < 0.05) (**Supplementary Figure 6A**). Collectively, these *in vitro* experiments demonstrate that IGFBP1 is overexpressed in ESCA cell lines and functions as an oncogenic regulator: it promotes the proliferative and invasive capabilities of ESCA cells, as well as regulates cell-cycle.

**Figure 11:**
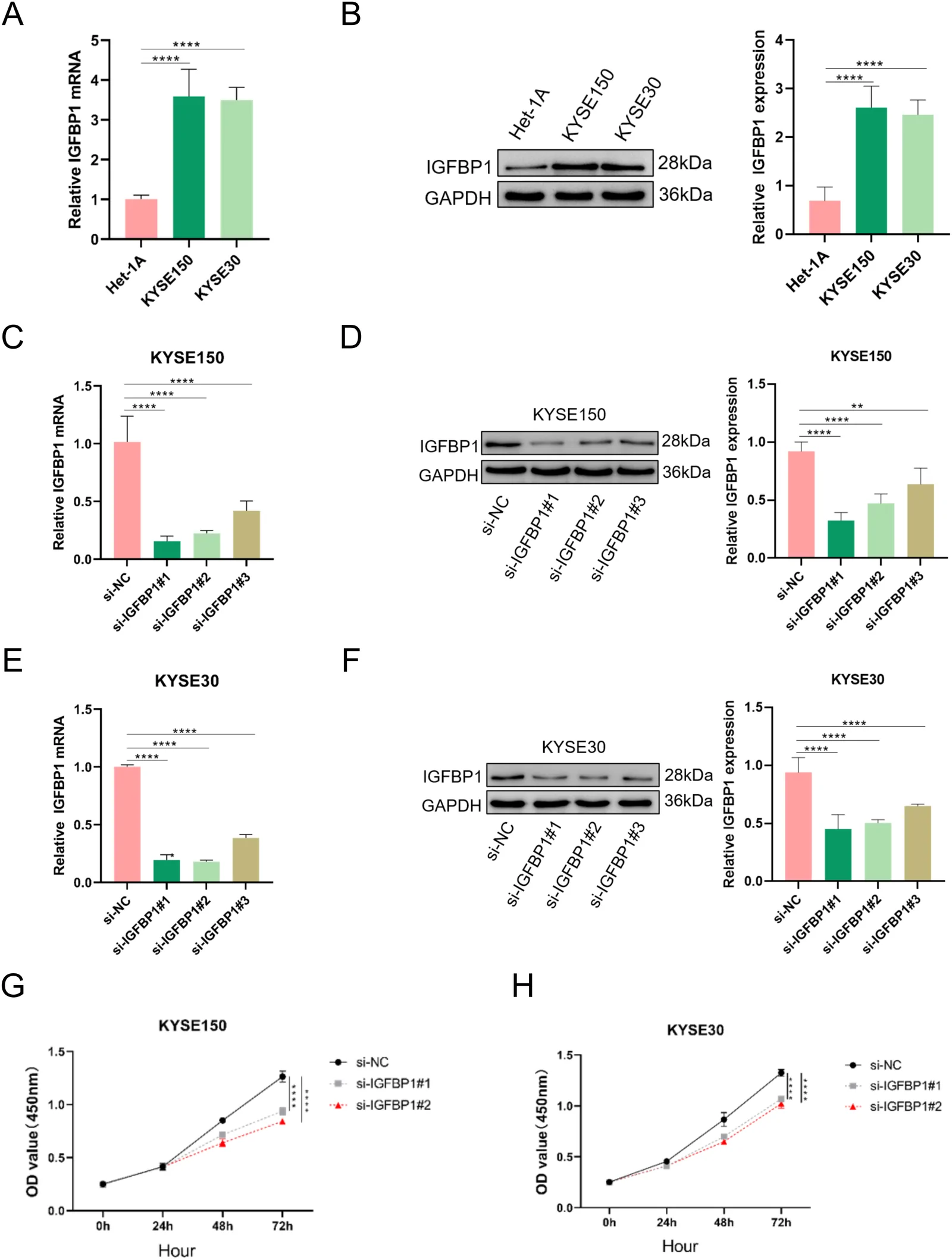
Expression of IGFBP1 *in vitro*. **(A)** qRT-PCR. **(B)** Western blot. **(C)** IGFBP1 knockdown efficiency in KYSE150 cells. **(D)** IGFBP1 protein knockdown efficiency in KYSE150 cells. **(E)** IGFBP1 mRNA knockdown efficiency in KYSE30 cells. **(F)** IGFBP1 protein knockdown efficiency in KYSE30 cells. **(G)** CCK-8 assay in KYSE150 cells. **(H)** CCK-8 assay in KYSE30 cells.

**Figure 12:**
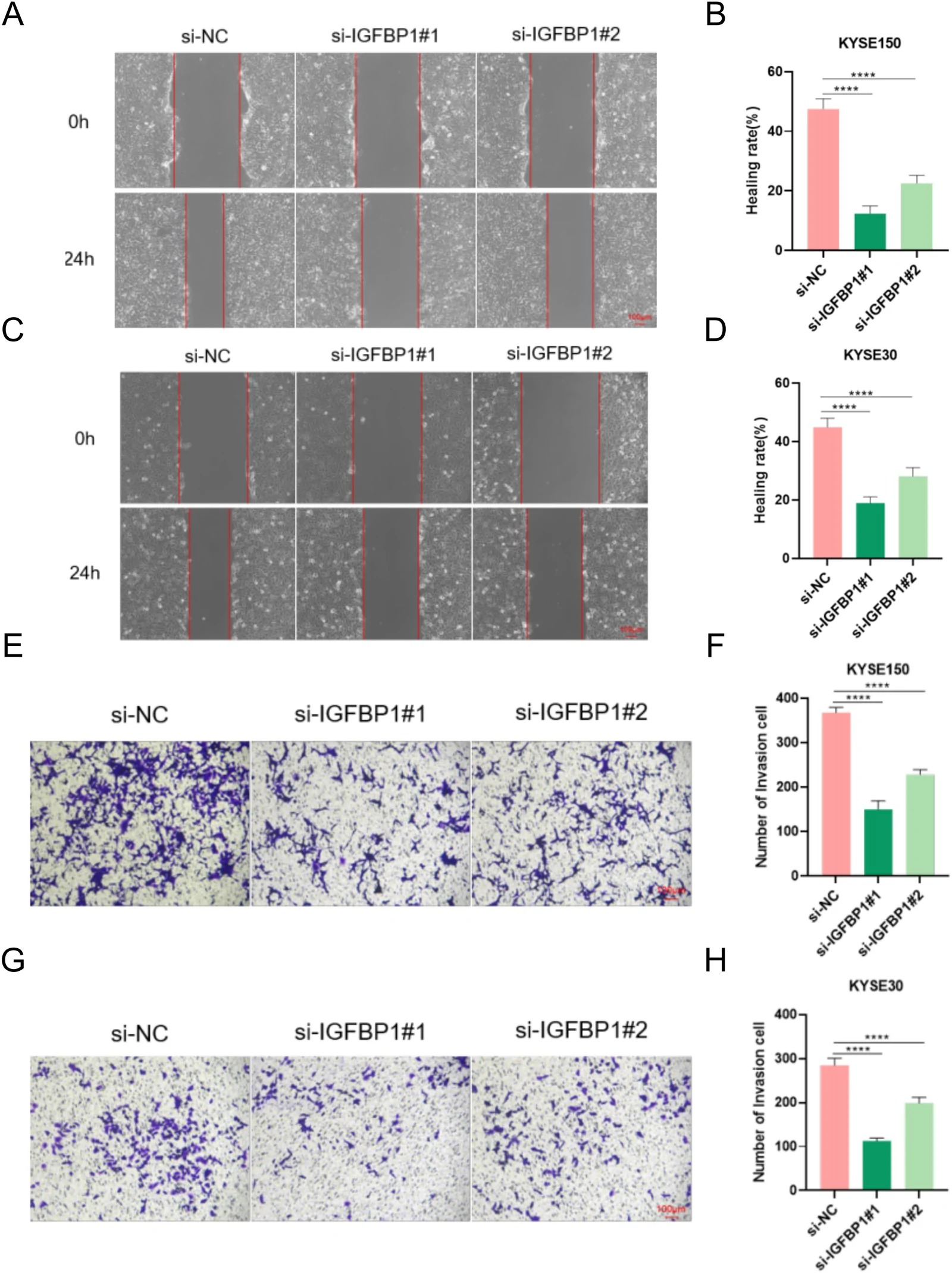
Effects of silencing IGFBP1 in ESCA cell migration and invasion. **(A)** Wound healing assay of KYSE150 cells transfected with si-IGFBP1#1, or si-IGFBP1#2. **(B)** Quantification of healing rate. **(C)** Wound healing assay of KYSE30 cells. **(D)** Quantification of healing rate. **(E)** Transwell invasion assay of KYSE150 cells. **(F)** Quantification of invaded cell number. **(G)** Transwell invasion assay of KYSE30 cells. **(H)** Quantification of invaded cell number.

### *In vivo* validation of the role of IGFBP1 in ESCA

To further validate the role of IGFBP1 in ESCA *in vivo*, we established xenograft models using KYSE150 and KYSE30 cells with IGFBP1 knockdown (sh-IGFBP1) or vector control. The tumors derived from sh-IGFBP1 cells (both KYSE150 and KYSE30) were significantly smaller than those from vector control cells (**Figure 13**). For KYSE150 xenografts, indicated significant difference in body weight between the vector and sh-IGFBP1 groups, and tumor volume and tumor weight demonstrated that IGFBP1 knockdown markedly inhibited tumor growth and reduced tumor weight (**Figure 13B**). Similarly, in KYSE30 xenografts, **Figure 13** showed body weight changes, and tumor volume (**Figure 13**) and tumor weight (**Figure 13**) confirmed that IGFBP1 knockdown significantly suppressed tumor growth. These *in vivo* data confirm the oncogenic function of IGFBP1 in ESCA, demonstrating that IGFBP1 promotes tumor growth in xenograft models—consistent with our *in vitro* findings and supporting IGFBP1 as a potential therapeutic target for ESCA.

**Figure 13:**
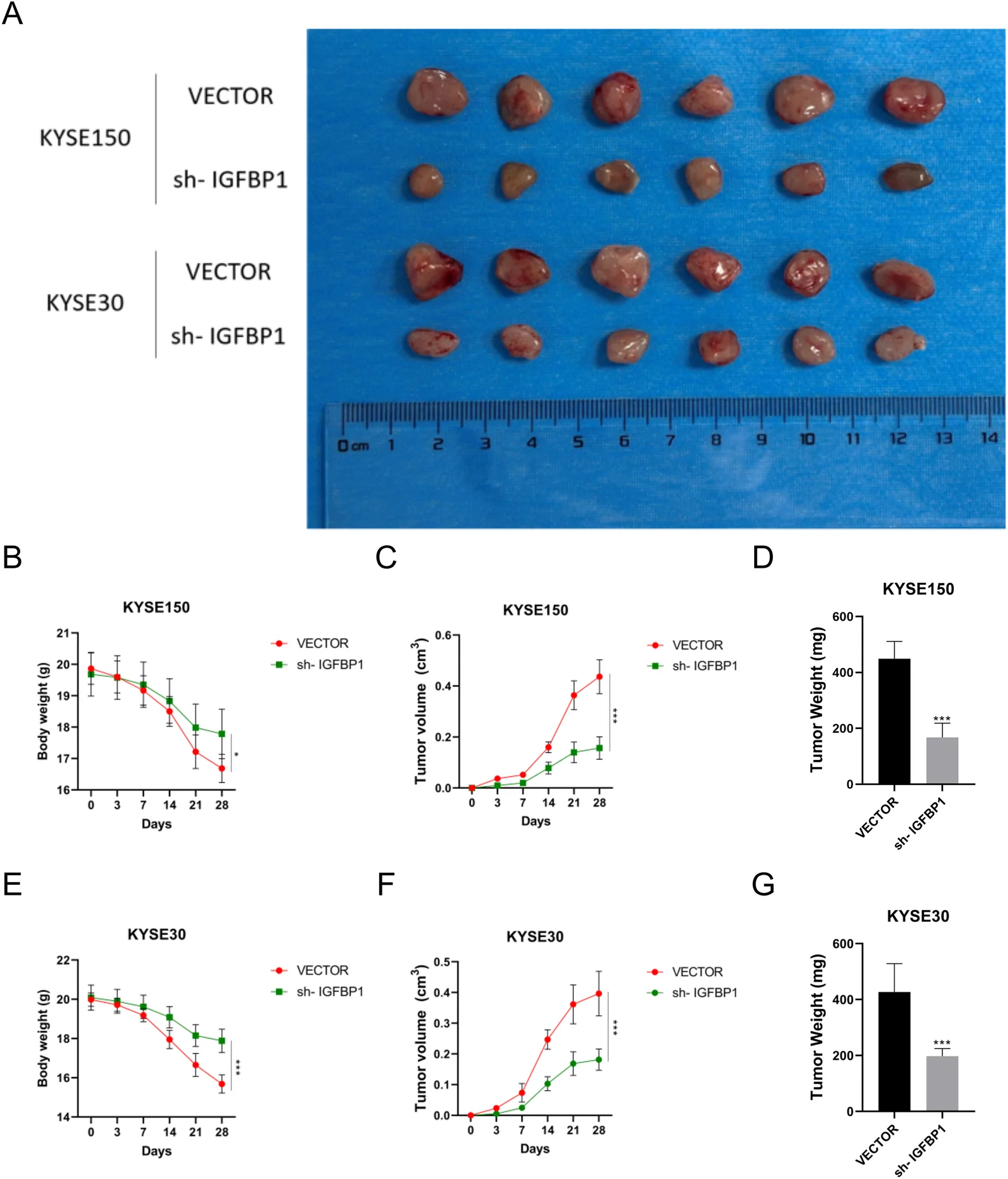
*In vivo* validation of IGFBP1 in ESCA. **(A)** Representative images of xenograft tumors from KYSE150 and KYSE30 cells (vector or sh-IGFBP1 groups). **(B)** Weight. **(C)** Tumor volume. **(D)** Tumor weight. **(E)** Body weight. **(F)** Tumor volume. **(G)** Tumor weight.

## Discussion

Esophageal carcinoma (ESCA) remains a global health burden with escalating incidence and persistently poor prognosis (26). Risk factors including tobacco use, alcohol consumption, and genetic susceptibility drive its pathogenesis, while the lack of reliable biomarkers leads to frequent late-stage diagnosis and suboptimal therapeutic outcomes (27). With a five-year survival rate below 20%, and marked histological heterogeneity between ESCC and EA, there is an urgent need for molecular markers that enable early detection and guide precision therapy (28).

IGFBP1 has emerged as a multifaceted regulator in tumor biology, though its role in ESCA has been insufficiently understood (29). In hepatocellular (30) and gastric cancers (31), IGFBP1 modulates metastasis and chemoresistance; in colorectal cancer, it interacts with tumor stroma to promote angiogenesis. However, existing ESCA studies have focused primarily on serum IGFBP1 levels, leaving tissue-specific expression patterns and mechanistic contributions largely unexplored. Our findings bridge this knowledge gap by demonstrating significant IGFBP1 upregulation in ESCA (p < 0.01), establishing its tissue-level relevance.

Notably, we further constructed and validated a clinicopathological diagnostic model incorporating IGFBP1 expression and key clinical variables, which exhibited excellent discriminatory ability (AUC = 0.884) and favorable clinical net benefit in both training and validation cohorts. Comparative analysis using a MLP model yielded comparable predictive performance, supporting the robustness of IGFBP1 as a stable and reliable diagnostic marker for ESCA.

Functional enrichment analyses revealed IGFBP1’s involvement in core oncogenic pathways. Gene Ontology terms clustered in protein-lipid complex remodeling, blood microparticle activity, and receptor-ligand interactions, supported by KEGG pathway enrichment in neuroactive ligand-receptor interaction and the PPAR signaling pathway. GSEA further identified protein containing complex remodeling (NES = 2.115, FDR < 0.001) and protein-lipid complex assembly (NES = 2.178, FDR < 0.001) as significantly activated in the IGFBP1-high cohort. Our WGCNA analysis further refined IGFBP1-associated gene networks, identifying a distinct co-expression module highly correlated with IGFBP1 in ESCA, with GSVA highlighting KRAS signaling as a potential pathway underlying IGFBP1-mediated oncogenic activity. Our single-cell analysis further pinpointed that IGFBP1 is predominantly expressed in smooth muscle cells (with negligible expression in other subsets) within the ESCA microenvironment, highlighting a cell-type-specific role of IGFBP1 in ESCA. These multi-omics insights preliminarily suggest that IGFBP1 may exert cell-type-specific roles in ESCA, potentially through regulating pathways associated with protein-lipid complex dynamics and neuroactive ligand-receptor interactions, with its smooth muscle cell-restricted expression implying a context-dependent functional relevance in the tumor microenvironment that warrants further validation.

Notably, our study uncovers IGFBP1’s immunosuppressive role in the ESCA microenvironment. Elevated IGFBP1 expression correlated with reduced Tcm cells, CD56dim cells and NK cells infiltration, shaping an immunologically “cold” tumor phenotype. CIBERSORT analysis further confirmed in IGFBP1-high cohort decreased infiltration of anti-tumor subsets: resting macrophages M1, resting dendritic cells and resting mast cells. Collectively, these ssGSEA and CIBERSORT-derived observations provide correlative evidence for IGFBP1-mediated immunosuppressive remodeling of the ESCA tumor microenvironment, with definitive mechanistic links awaiting validation through targeted functional experiments.

Emerging evidence implicates m6A modification in regulating cancer-associated genes, with IGFBP1 emerging as a key m6A-related protein that exerts critical regulatory functions in tumorigenesis and progression (32). Our analysis revealed positive co-expression between IGFBP1 and m6A readers (IGF2BP1, r = 0.218; IGF2BP3, r = 0.233), and negative co-expressions with ALKBH5, YTHDF2, FTO, METTL3, METTL14 and YTHDC1. Collectively, these correlative findings hint at a potential interplay between IGFBP1 and the m^6^A modification landscape in ESCA, which may underpin the epigenetic modulation of oncogenic signaling cascades, which merits further functional exploration (e.g., m6A-seq to identify IGFBP1-targeted transcripts).

While the prognostic value of IGFBP1 has been previously documented in various tumor types, we show that IGFBP1 overexpression predicted reduced OS and DSS in ESCA. Our further analyses also revealed close correlations between IGFBP1 expression and patient demographics: age (>60), gender (male), histological type (adenocarcinoma), clinical T stage (T3&T4), weight (>70), BMI (>25), smoker (yes), alcohol history (no) and radiation therapy (no). Collectively, these findings highlight IGFBP1 as a promising prognostic indicator that stratifies ESCA patients into distinct risk subgroups based on both survival endpoints and clinically relevant demographic and pathological features.

Our cellular and animal experiments collectively establish a pro-oncogenic function of IGFBP1 in ESCA progression, aligning with its previously reported prognostic significance. In esophageal epithelial cells, the elevated expression of IGFBP1 in KYSE150 and KYSE30 lines relative to the normal Het-1A cells points to a potential association between IGFBP1 upregulation and the malignant transformation of esophageal epithelial cells. Functional knockdown confirmed that IGFBP1 depletion curtails ESCA cell proliferative, and invasive abilities in cellular context, concurrent with a notable G2/M cell cycle arrest. These observations imply that IGFBP1 may facilitate tumor aggressiveness by modulating cell cycle progression and motility-related cytoskeletal dynamics (aligning with our GSEA findings in protein-lipid complex pathways). Xenograft models reinforced these phenotypes, as shRNA-mediated IGFBP1 silencing resulted in a marked decrease in tumor volume/weight without causing any significant alterations in host body weight, signifying that the tumor-suppressive effects are not attributable to systemic toxicity.

Limitations of the current study include: (1) bioinformatic analyses constrained by the TCGA-ESCA cohort’s sample size (limiting power to detect subtle correlations with ESCA molecular subtypes); (2) further mechanistic exploration of IGFBP1’s upstream regulators and downstream signaling. Notably, the multiple bioinformatics analyses adopted in this study, including WGCNA, GO/KEGG enrichment, GSEA, single-cell analysis, in silico knockdown, and GSVA, rely on distinct data sources and analytical workflows, leading to divergent analytical conclusions across different assays, and all relevant findings are merely based on computational predictions and expression correlation analysis without verifying direct regulatory relationships and definite causal mechanisms. Nevertheless, although our findings identify IGFBP1 as a promising biomarker for ESCA, the optimal clinical application strategy of this biomarker remains undefined. Moreover, tissue-specific IGFBP1 expression exerts a more critical prognostic and regulatory role in ESCA progression, which requires systematic verification and in-depth investigation in future independent studies. Future research should explore IGFBP1-targeted therapies (e.g., in combination with immune checkpoint inhibitors or m6A modulators) which may transform ESCA management.

## Conclusion

In conclusion, this study positions IGFBP1 as a multifunctional biomarker in ESCA pathogenesis, influencing cell cycle dynamics, immune evasion, and epigenetic regulation. Together, these results underscore IGFBP1 as a functionally critical ESCA driver, providing a mechanistic rationale for its prognostic utility as a prognostic biomarker in stratifying ESCA patients into clinically relevant risk subgroups.

## Data Availability

The original contributions presented in the study are included in the article/**Supplementary Material**. Further inquiries can be directed to the corresponding authors.
