# Supplementary material for: IGFBP1 is a prognostic biomarker in esophageal carcinoma: mechanistic insights from bioinformatics profiling to in vitro functional validation
Source: Front Immunol. 2026 Sep 16;17:1836824. doi: 10.3389/fimmu.2026.1836824 (PMC13624076; doi:10.3389/fimmu.2026.1836824)
Supplement: Supplementary file 1 [file Table1.docx]

**Supplementary Materials**

**Supplementary Figures**

**Supplementary Figure 1.**

**Supplementary Figure 1. Expression of IGFBP1 in ESCA tissue microarray.** (A) ESCA tissue microarray. (B) Repsentative normal and cancer tissues. (C) Nuclear staining intensity. (D) Membrane staining intensity. (E) Cytoplasmic staining intensity.

**Supplementary Figure 2.**

**Supplementary Figure 2. Single‑cell expression and correlation analysis of IGF family members in ESCA.** UMAP visualization showing the spatial distribution and expression levels of (A) IGF1, (B) IGF2, (C) IGF1R, and (D) IGF2R. Violin plots quantifying the expression levels of (E) IGF1, (F) IGF2, (G) IGF1R, and (H) IGF2R. Scatter plots showing the correlation between IGFBP1 expression and the expression of (I) IGF1, (J) IGF2, (K) IGF1R, and (L) IGF2R, with correlation coefficients (R) and p-values indicated.

**Supplemetatry Figure 3.**

**Supplementary Figure 3. Correlation between IGFBP1 expression and immune cell infiltration in ESCA.** Enrichment scores of, (A) Eosinophils, (B) Th17 cells, (C) pDC. (D) Eosinophils. (E) Th17 cells. (F) pDC. * p < 0.05

**Supplemetatry Figure 4.**

**Supplementary Figure 4. Correlation between IGFBP1 expression and immune cell infiltration in ESCA.** (A) Correlation heatmap of IGFBP1 with 24 immune cell types. (B) Macrophages M1. (C) Dendritic cells resting. (D) Mast cells resting. (E) Macrophages M1. (F) Dendritic cells resting. (G) Mast cells resting. * p < 0.05, ** p < 0.01

**Supplemetatry Figure 5.**

**Supplementary Figure 5. DNA methylation of IGFBP1 in ESCA.** (A) Association between IGFBP1 expression and methylation levels. (B) Promoter methylation level of IGFBP1 in ESCA. (C) Kaplan-Meier curves for IGFBP1 sites at cg00110785. (D) Kaplan-Meier survival curves for IGFBP1 at cg05660795.

**Supplemetatry Figure 6.**

**Supplementary Figure 6. Mutation status of IGFBP1 in ESCA.** (A) Clinical/genomic feature summary (mutation, CNV, prognosis) of IGFBP1 in ESCA. (B) Overall survival. (C) Disease specific survival. (D) Disease free survival. (E) Progression free survival.

**Supplemetatry Figure 7.**

**Supplementary Figure 7. Prognostic value of IGFBP1 in various ESCA subtypes.** (A) Age: >60. (B) Gender: Male. (C) Histological type: Adenocarcinoma. (D) Clinical T stage: T3&T4. (E) Weight: >70. (F) BMI: >25. (G) Smoker: Yes. (H) Alcohol history: No. (I) Radiation therapy: No.

**Supplemetatry Figure 8.**

**Supplementary Figure 8. Effects of IGFBP1 knockdown on cell cycle distribution in ESCA cells.** (A, B) Cell cycle analysis of KYSE150 cells transfected with si-NC, si-IGFBP1#1, or si-IGFBP1#2. (A) Representative flow cytometry plots. (B) Quantification of cell percentage in G0/G1, S, and G2/M phases. (C) Representative flow cytometry plots. (D) Quantification of cell percentage in G0/G1, S, and G2/M phases. ****P < 0.0001, ***P < 0.001.

**Supplementary Table 1. Logistic regression analysis of IGFBP1 in ESCA.**

| Characteristics | Total (N) | OR (95% CI) | P value |
| --- | --- | --- | --- |
| Age (> 60 vs. <= 60) | 163 | 0.692 (0.373 - 1.283) | 0.242 |
| Gender (Male vs. Female) | 163 | 1.416 (0.582 - 3.444) | 0.443 |
| Clinical T stage (T3&T4 vs. T1&T2) | 52 | 4.843 (1.176 - 19.950) | **0.029** |
| Clinical N stage (N1&N2 vs. N0) | 43 | 2.057 (0.562 - 7.528) | 0.276 |
| Clinical M stage (M1 vs. M0) | 52 | 4.167 (0.960 - 18.081) | 0.057 |
| Clinical stage (Stage III&Stage IV vs. Stage I&Stage II) | 54 | 2.615 (0.858 - 7.970) | 0.091 |
| Weight (> 70 vs. <= 70) | 161 | 2.986 (1.571 - 5.675) | **< 0.001** |
| BMI (> 25 vs. <= 25) | 154 | 1.711 (0.902 - 3.249) | 0.100 |
| Histological type (Squamous Cell Carcinoma vs. Adenocarcinoma) | 163 | 0.195 (0.100 - 0.381) | **< 0.001** |
| Barretts esophagus (Yes vs. No) | 133 | 1.786 (0.732 - 4.360) | 0.203 |
| Tumor cental location (Distal vs. Proximal&Mid) | 162 | 2.507 (1.247 - 5.042) | **0.010** |
| Radiation therapy (Yes vs. No) | 145 | 0.296 (0.131 - 0.672) | **0.004** |
| Reflux history (Yes vs. No) | 137 | 1.887 (0.934 - 3.812) | 0.077 |
