# Supplementary material for: IGFBP1 is a prognostic biomarker in esophageal carcinoma: mechanistic insights from bioinformatics profiling to in vitro functional validation
Source: Front Immunol. 2026 Sep 16;17:1836824. doi: 10.3389/fimmu.2026.1836824 (PMC13624076; doi:10.3389/fimmu.2026.1836824)
Supplement: Supplementary file 2 [file DataSheet1.zip › 喀什大学实验动物福利伦理审查意见书.pdf]

[illegible]

喀什大学实验动物福利伦理审查意见书

Kashi University Ethical Review Opinion on Animal Welfare in Animal Experimentation

合规性声明：本伦理委员会的组成和运行遵循中国相关法律法规，审查和工作过程不受伦理委员会以外的任何组织及个人的影响。

Compliance Statement: The composition and operation of this Ethics Committee comply with relevant Chinese laws and regulations. The review and work processes are not influenced by any organization or individual outside the Ethics Committee.

批准编号 Approval No	KSU-AEC-A-2025-002
项目名称 Project Title	IGFBP1 作为食管癌预后标志物：从生物信息学分析到体外功能验证的机制研究<br>IGFBP1 is a prognostic biomarker in esophageal carcinoma: mechanistic insights from bioinformatics profiling to in vitro functional validation
申报单位 Applicant Unit	喀什大学医学院
项目负责人 Project Leader	陈海林
动物品种 Animal Strain	BALB/c 裸鼠
动物数量 Animal Quantity	24 只
审查结果 Review Result	符合动物福利伦理要求，同意实验<br>welfare ethical requirements, experiment approved

扫描全能王 创建

审查意见：

Review Opinion

根据动物实验伦理原则，本实验经喀什大学实验动物福利伦理委员会审议，符合伦理要求，同意此项研究在相应的设施区域进行实验。

In accordance with the ethical principles of animal experimentation, this study has been reviewed by the Kashi University Laboratory Animal Welfare and Ethics Committee. It meets the ethical requirements, and approval is granted to conduct the study in appropriate facility areas.

主任委员签字：[illegible]

日期 Date ： 2025.10.1

喀什大学实验动物福利伦理委员会（盖章）

地址：新疆喀什市学院路 29 号 邮编：844000 电话：0998-2899203

扫描全能王 创建
