# Supplementary material for: IGFBP1 is a prognostic biomarker in esophageal carcinoma: mechanistic insights from bioinformatics profiling to in vitro functional validation
Source: Front Immunol. 2026 Sep 16;17:1836824. doi: 10.3389/fimmu.2026.1836824 (PMC13624076; doi:10.3389/fimmu.2026.1836824)
Supplement: Supplementary file 2 [file DataSheet1.zip › 伦理审查报告-李倩男.pdf]

上海卓立生物科技有限公司伦理委员会

科研组织芯片使用伦理审查意见

<table>
<tr><td>受理号</td><td>LLSM-15-01</td><td>意见号</td><td colspan="3">LLSM-15-01-240805</td></tr>
<tr><td>研究项目名称</td><td colspan="5">IGFBP1作为食管癌预后标志物：从生物信息学分析到体外功能验证的机制研究</td></tr>
<tr><td>申请单位</td><td>喀什地区第一人民医院</td><td>主要研究者：</td><td>艾尼瓦尔江·阿布都肉苏力</td><td>承提责任</td><td>主要研究者☑</td></tr>
<tr><td>研究分类</td><td colspan="5">利用组织芯片进行观察性研究和回顾性分析</td></tr>
<tr><td>组织芯片来源</td><td colspan="5">委托方芯片库</td></tr>
<tr><td>审查文件清单</td><td colspan="5">1、 临床科研初始研究审查表
2、 研究方案
3、 知情同意书
4、 研究人员列表</td></tr>
<tr><td>审查意见</td><td colspan="5"></td></tr>
<tr><td>有效期</td><td colspan="5">一年 （使用完后芯片需要寄回样本库中心：上海市闵行区江月路999号9号楼4F，上海卓立生物科技有限公司）</td></tr>
<tr><td>审查声明</td><td colspan="5">兹证明本研究的设计和方法符合国家卫计委《涉及人的生物医学研究伦理审查办法(2016)》、国家药监局、卫健委《药物临床试验质量管理规范(2020)》、WMA《赫尔辛基宣言》和CIOMS《人体生物医学研究国际道德指南》的伦理原则，批准同意喀什地区第一人民医院主要研究者艾尼瓦尔江·阿布都肉苏力关于IGFBP1作为食管癌预后标志物：从生物信息学分析到体外功能验证的机制研究项目使用样本库食管癌组织芯片 ZL-EsoS961 作为研究材料。

主任委员（签名）：

上海卓立生物科技有限公司 伦理委员会

2026年02月14日</td></tr>
</table>
